# Construction of lncRNA-related competing endogenous RNA network and identification of hub genes in recurrent implantation failure

**DOI:** 10.1186/s12958-021-00778-1

**Published:** 2021-07-09

**Authors:** Jialyu Huang, Ning Song, Leizhen Xia, Lifeng Tian, Jun Tan, Qianqian Chen, Jing Zhu, Qiongfang Wu

**Affiliations:** 1 Reproductive Medical Center, Jiangxi Provincial Maternal and Child Health Hospital, 330006 Nanchang, China; 2 Department of Histology, Embryology, Genetics and Developmental Biology, Shanghai Key Laboratory of Reproductive Medicine, Shanghai Jiao Tong University School of Medicine, 200025 Shanghai, China; 3 Reproductive Medical Center, The First Affiliated Hospital of Wenzhou Medical University, 325000 Wenzhou, China

**Keywords:** Recurrent implantation failure, Long non-coding RNA, Competing endogenous RNA, Weighted gene co-expression network analysis

## Abstract

**Background:**

The mechanism of recurrent implantation failure (RIF) is unclear at present and poor endometrial receptivity may be one of the leading reasons. This study aims to construct a competing endogenous RNA (ceRNA) network and identify potential hub genes underlying the development of RIF.

**Methods:**

Weighted gene co-expression network analysis was performed based on differentially expressed mRNAs (DEMs) and lncRNAs (DELs) from the GSE111974 dataset. Functional enrichment analyses of gene modules were conducted using Gene Ontology classification and Kyoto Encyclopedia of Genes and Genomes pathway. A lncRNA-miRNA-mRNA ceRNA regulatory network was constructed according to predictive interaction derived from the LncRNADisease, miRTarBase, miRDB and TargetScan databases. Topological analysis determined the key genes with the highest centroid and their expressions were further verified using public datasets and quantitative real-time polymerase chain reaction.

**Results:**

A total of 1500 DEMs and 3 DELs were significantly up-regulated, whereas 1022 DEMs and 4 DELs were significantly down-regulated in the RIF group compared with the control group. Six functional co-expression modules were enriched in various biological processes, such as cell adhesion, regulation of cell motility and cellular response to vascular endothelial growth factor stimulus. Five hub genes were identified in the ceRNA network, of which GJA1 was down-regulated whereas TET2, MAP2K6, LRRC1 and TRPM6 were up-regulated in RIF endometrium.

**Conclusions:**

We constructed a lncRNA-associated ceRNA network and identified five novel hub genes in RIF. This finding could be helpful to understand the molecular mechanism for RIF pathogenesis, and may provide novel insights for its early diagnosis and treatment.

**Supplementary Information:**

The online version contains supplementary material available at 10.1186/s12958-021-00778-1.

## Background

Successful implantation is a process which requires a synchronized and coordinated interaction between the embryo and the endometrium [1]. Over the past decades, *in vitro* fertilization-embryo transfer (IVF-ET) has become an effective approach for infertility treatment with the improvements in ovarian stimulation and laboratory procedures [2]. Nevertheless, for each IVF-ET cycle, the rates of implantation and livebirth still remain modest at best [3]. In addition, a significant proportion of couples would experience recurrent implantation failure (RIF), leading to great financial and psychological burden in these patients [4].

The definition of RIF is varied and no consensus has been reached yet [5]. According to the latest criteria proposed by Coughlan et al. [6], RIF refers to the failure to achieve clinical pregnancy after transfer of ≥ 4 morphologically good-quality embryos for ≥ 3 fresh or frozen-thawed cycles in women less than 40 years old. Recent evidences suggest that RIF may be mainly associated with poor endometrial receptivity and several molecular changes have been detected in women with RIF, such as mucin 1, integrin β3, homeobox A10 and leukemia inhibitor factor [5, 7–10]. However, the precise etiology and pathogenesis of RIF have not been fully revealed. Further studies are still urgently needed to elucidate the underlying mechanism, identify new prognostic biomarkers and develop potential therapeutic targets.

With the advances in high-throughput sequencing technology, it is now well-established that the regulation of biological processes not only relies on protein-coding messenger RNA (mRNA), but also on non-coding RNA such as micro RNA (miRNA), circular RNA and long non-coding RNA (lncRNA) [11]. LncRNA, located in the cell nucleus and/or cytoplasm, is > 200 nucleotides in transcript length [12]. By competing for and binding to shared miRNAs, lncRNAs can regulate the expression of other mRNAs, which is called competing endogenous RNA (ceRNA) network [12]. At present, the ceRNA network has been proven to be involved in the development of various diseases, including cancer [13], gestational diabetes [14], systemic lupus erythematosus [15] and polycystic ovary syndrome [16]. Moreover, accumulating studies show that specific mRNAs, miRNAs and lncRNAs are differentially expressed in the secretory endometrium of RIF women, indicating their involvement in endometrial receptivity defects [17–21]. Nonetheless, the regulatory mechanism of the lncRNA-miRNA-mRNA ceRNA network in RIF is still poorly investigated and remains largely unclear so far [22, 23].

In the present study, we obtained RIF-related expression profiles of mRNAs and lncRNAs from the Gene Expression Omnibus (GEO) database. The co-expression modules and ceRNA network were constructed following integrated bioinformatic analyses, with novel hub genes identified and validated as potential targets for the pathogenesis of RIF.

## Methods

### Data collection and pre-processing

Series matrix files of three datasets (GSE111974, GSE71835 and GSE103465) were downloaded from the GEO database (http://www.ncbi.nlm.nih.gov/geo/). The platforms were based on GPL17077 (Agilent-039494 SurePrint G3 Human GE v2 8 × 60 K Microarray 039381), GPL10558 (Illumina HumanHT-12 V4.0 expression bead chip) and GPL15207 (GeneChip® PrimeView™ Human Gene Expression Array), respectively. All data were processed by the R software (version 3.4.0; https://www.r-project.org/) and normalized by Robust Multichip Average (RMA) algorithms. GSE111974 was used to screen the key genes related to RIF [24], whereas GSE71835 and GSE103465 were utilized for verification [25, 26].

### Identification of differentially expressed mRNAs (DEMs) and lncRNAs (DELs)

The limma package was used for identifying DEMs and DELs between the RIF samples and matched normal samples in GSE111974 [27]. An adjusted *P* < 0.05 and an absolute log2 fold change (|log2FC|) > 1 were considered statistically significant. According to the similarity of lncRNAs and mRNAs expression level, two-way hierarchical clustering and heatmap illustration were performed by the heatmap package in R software to classify samples into different groups and reveal the relationship between different samples.

### Construction of co-expression network

Gene co-expression networks were constructed based on DEMs and DELs using the weighted gene co-expression network analysis (WGCNA) package in R software [28]. Firstly, the outliers in the samples with limited expression information were checked and removed. Subsequently, the power of *β*-value was introduced to transform the similarity matrix into an adjacency matrix. For the best scale-free fit index and average connectivity, the soft thresholding value (*β*) was determined to be 14 in the present study. On this basis, the topological overlap matrix (TOM) and the corresponding dissimilarity values (1-TOM) were further calculated [29]. Ultimately, genes with highly absolute correlations were clustered into the same module to generate a cluster dendrogram by The Dynamic Tree Cut method. The minimum number of genes *per* module was pre-set to be 10.

### Functional enrichment analysis

To further analyze the potential biological processes, cellular components, molecular functions and pathways of the gene modules, the Gene Ontology (GO) and Kyoto Encyclopedia of Genes and Genomes (KEGG) analyses were performed using the Database for Annotation, Visualization and Integrated Discovery (DAVID, https://david.ncifcrf.gov/) [30]. GO terms and KEGG pathways were classified as enriched when *P* < 0.05.

### Construction of ceRNA network

To construct the ceRNA network, we obtained the predictive interaction of DELs in the WGCNA modules with miRNAs from the LncRNADisease database (http://www.rnanut.net/lncrnadisease/) [31]. Meanwhile, the interaction between DEMs and miRNAs were downloaded and validated using the following three databases: miRTarBase (http://miRTarBase.cuhk.edu.cn/) [32], miRDB (http://www.mirdb.org/) [33] and TargetScan (http://www.targetscan.org/) [34]. A lncRNA-miRNA‐mRNA ceRNA network was constructed and visualized based on lncRNA‐miRNA and miRNA‐mRNA pairs using the Cytoscape software (version 3.7.0; Cytoscape Consortium, USA).

### Identification of hub genes

The mRNAs in the intersection of the WGCNA network and the ceRNA network were considered to play key roles. These mRNAs were re-mapped into the WGCNA network and we then assessed the topological property of each node in the interaction network by calculating three parameters: degree centrality (DC), betweenness centrality (BC) and closeness centrality (CC). Generally, a larger quantitative value indicates a greater significance of the node in the network. The MATLAB Z-score function was used to standardize these parameters and the formula was as follows: Z-score = (x–mean(x))/std(x), where x was the value of the topological parameter, mean (x) denoted the mean value and std (x) represented the standard deviation [35]. After summing the DC, BC and CC Z-scores of each node, we selected the top 5 genes as the hub genes in the network.

### Validation of hub lncRNAs and genes

To verify the differential expression of hub lncRNAs and genes in RIF patients, expression data were further extracted from the GSE71835 and GSE103465 datasets. The Wilcoxon test was performed for between-group comparison. The level of statistical significance was set at *P* < 0.05 on two-sided test.

### Sample collection

Endometrial tissues were obtained from patients who underwent IVF-ET treatment at the Reproductive Medical Center, the First Affiliated Hospital of Wenzhou Medical University. Specifically, pipe suction curettage (Runting, Soochow, China) was used to collect mid-secretory endometrium in 7 days after ovulation during natural menstrual cycles. The RIF group consisted of 10 women that failed to conceive after 3 or more transfer cycles with at least 4 morphologically high-grade embryos [6]. The control group included another 10 women that achieved clinical pregnancy in their first or second embryo transfer. All women were less than 40 years old, exhibited regular ovulation and normal body mass index, and had no hormonal therapy in the last 3 months. Exclusion criteria were as follows: congenital uterine malformations, uterine fibroids, endometrial polyps or hyperplasia, intrauterine adhesions, endometritis, endometriosis, adenomyosis, hydrosalpinx, polycystic ovary syndrome, hyperprolactinemia, thyroid dysfunction, thrombotic disorders, immune-related diseases, infectious diseases and abnormal parental karyotypes. All participants provided written informed consents and ethical approval was obtained from the Institutional Review Board of the First Affiliated Hospital of Wenzhou Medical University (No. 2019-07).

### RNA extraction and quantitative real-time polymerase chain reaction (qRT-PCR)

Total RNA was extracted from endometrial samples by TRIzol™ Reagent (Invitrogen, Carlsbad, USA) and reverse-transcribed into cDNA using PrimeScript™ RT Master Mix (TaKaRa, Dalian, China). The qRT-PCR was conducted in triplicates using TB Green™ Premix Ex Taq™ II (TaKaRa, Dalian, China) on the QuantStudio™ 6 Flex Real-Time PCR System (Applied Biosystems, Foster City, USA). Relative mRNA expression was analyzed by the 2^−△△Ct^ method and normalized to β-actin as the endogenous reference gene. The primer sequences of targeted genes are presented in Table S1.

### Statistical analysis

Quantitative variables were presented as mean ± standard deviation. After assessment of normality by the Shapiro-Wilk test, comparisons between the control and RIF groups were performed using the Student’s *t* test (for parametric data) or Mann-Whitney *U* test (for nonparametric data). For qualitative variables, data were described as number with proportion, and compared by χ^2^ test or Fisher’s exact test as appropriate. Differences were considered to be statistically significant for *P* < 0.05. All statistical analyses were conducted in SPSS 26.0 software (IBM Corp., Armonk, USA).

## Results

### Data acquisition

From the GSE111974 dataset, we downloaded expression profiles in the endometrial tissue samples of 24 patients with RIF and 24 normal women of childbearing age by RNA-sequencing. A total of 17,596 genes were obtained for analysis, including 17,154 mRNAs and 442 lncRNAs. In addition, the GSE71835 dataset included expression profiles of 6 RIF and 6 normal women, and the GSE103465 dataset had 3 samples in each respective group.

### Identification of DEMs and DELs

**Fig. 1:**
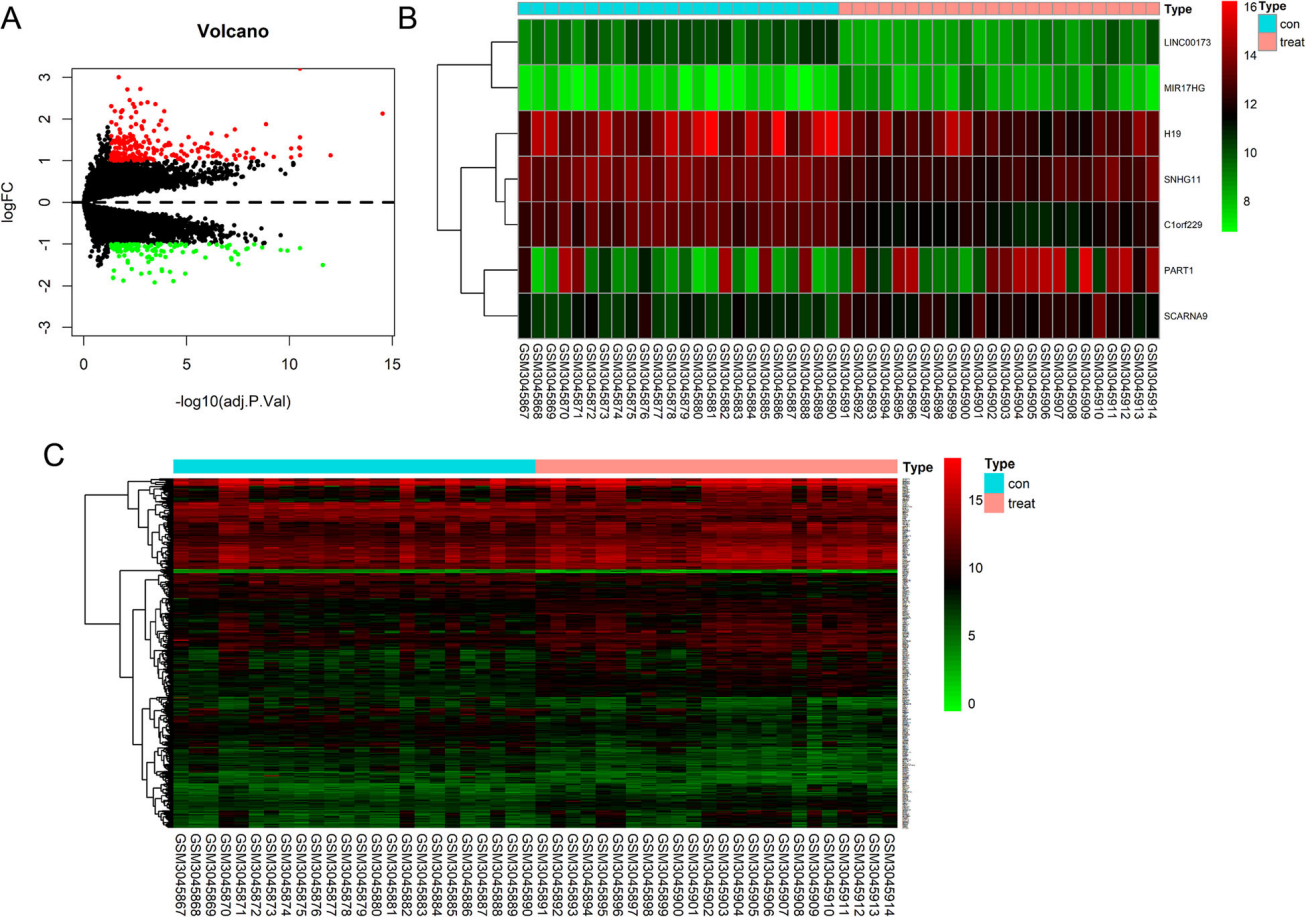
Identification of DEMs and DELs based on GSE111974 dataset. **A** Volcano plot of differentially expressed genes in endometria of RIF and control groups. The red dots represent significantly upregulated genes, and the green dots represent significantly downregulated genes. Hierarchical clustering heatmaps of (**B**) DELs and (**C**) DEMs in endometria of RIF and control groups. The color from green to red shows a trend from low expression to high expression. DEMs, differentially expressed mRNAs; DELs, differentially expressed lncRNAs; RIF, recurrent implantation failure

A total of 2522 DEMs and 7 DELs were identified from the GSE111974 dataset. Among them, 1500 DEMs and 3 DELs were significantly up-regulated, whereas 1022 DEMs and 4 DELs were significantly down-regulated in the RIF group compared with those in the normal group. The corresponding volcano plot and clustering heatmaps are shown in Fig. 1.

### Construction of co-expression network

**Fig. 2:**
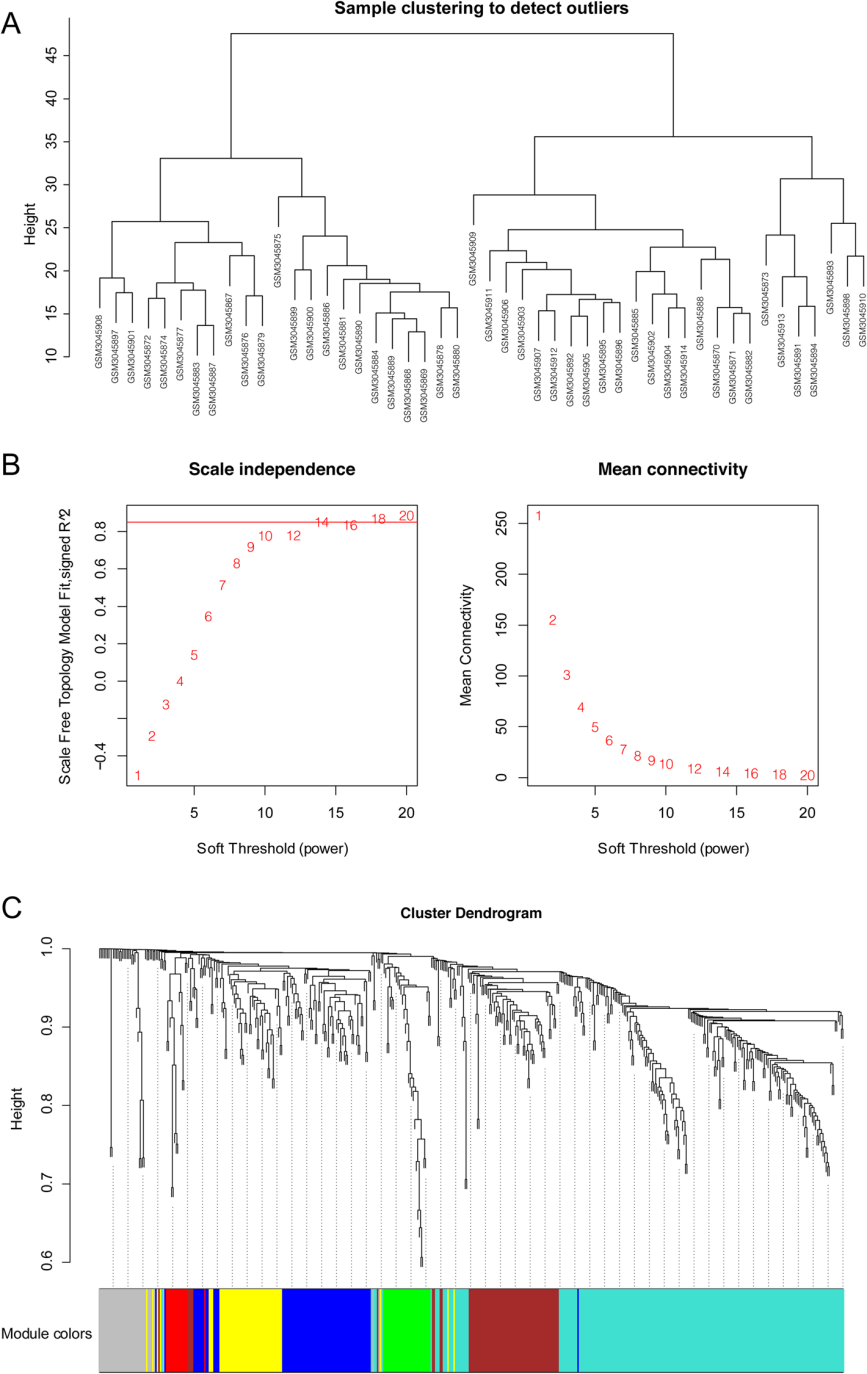
Weighted gene co-expression network analysis based on GSE111974 dataset. **A** Sample clustering to detect outliers. **B** Scale-free topology model fit (left) and mean connectivity (right) for determining the optimal soft threshold value. **C** Cluster dendrogram of genes. Each branch represents one gene and seven modules are displayed in different colors

**Fig. 3:**
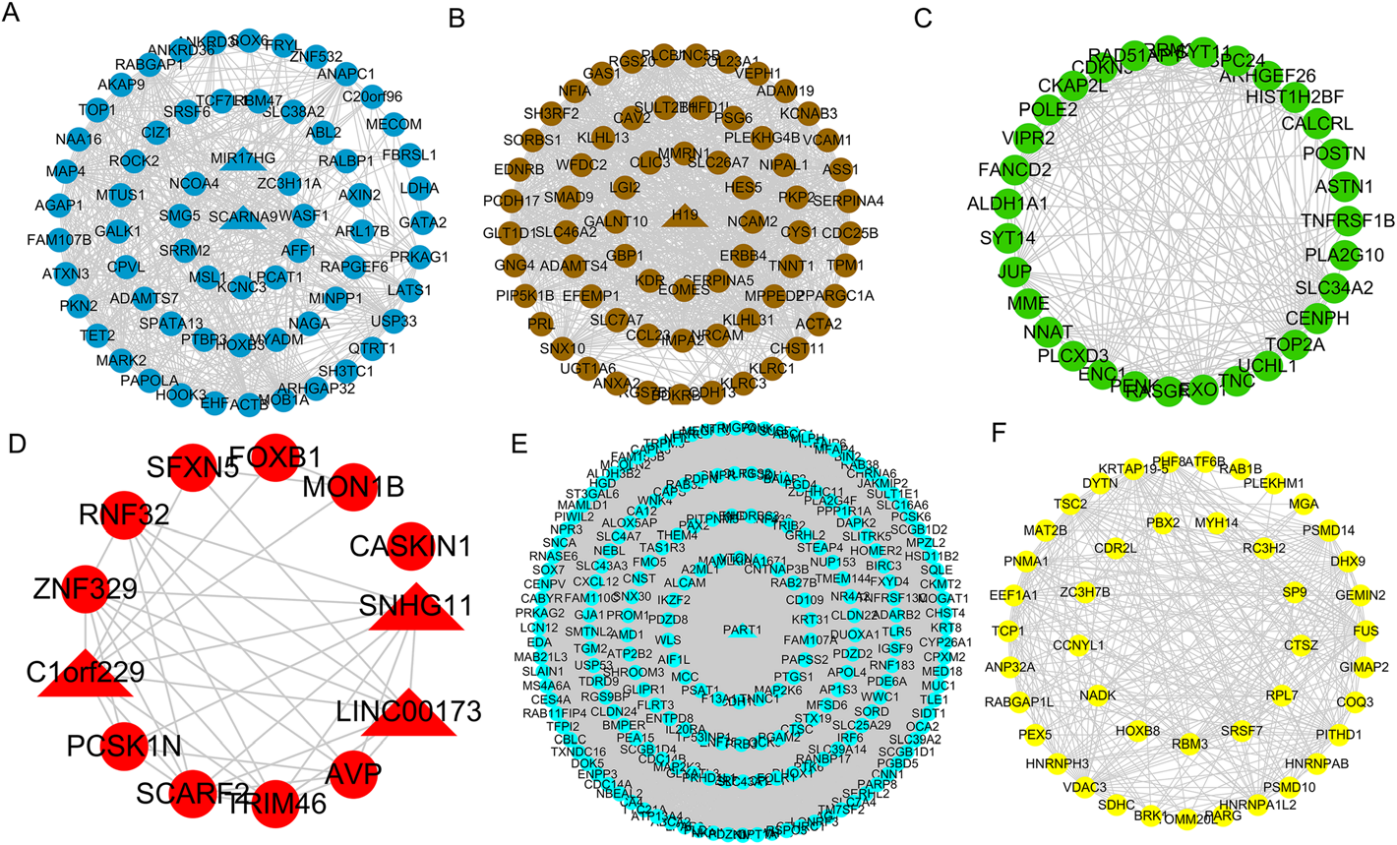
Co-expression networks of the six modules. **A** The blue module. **B** The brown module. **C** The green module. **D** The red module. **E** The turquoise module. **F** The yellow module. The round nodes represent mRNAs, the triangle nodes represent lncRNAs, and the edges between nodes indicate co-expressions

We identified seven co-expression modules using WGCNA, which revealed the molecular gene regulatory networks based on pairwise correlations between the differentially expressed RIF RNAs (Fig. 2). The RNAs that might function together were considered as one module and assigned with one color. The gray represented all genes that could not be incorporated into any module. As the remaining module network presented, the blue module included 63 mRNAs and 2 lncRNAs; the green module included 31 mRNAs; the brown module included 66 mRNAs and 1 lncRNA; the red module included 10 mRNAs and 3 lncRNAs; the yellow module included 44 mRNAs; and the turquoise module included 203 mRNAs and 1 lncRNA (Fig. 3).

### Functional enrichment analysis

**Fig. 4:**
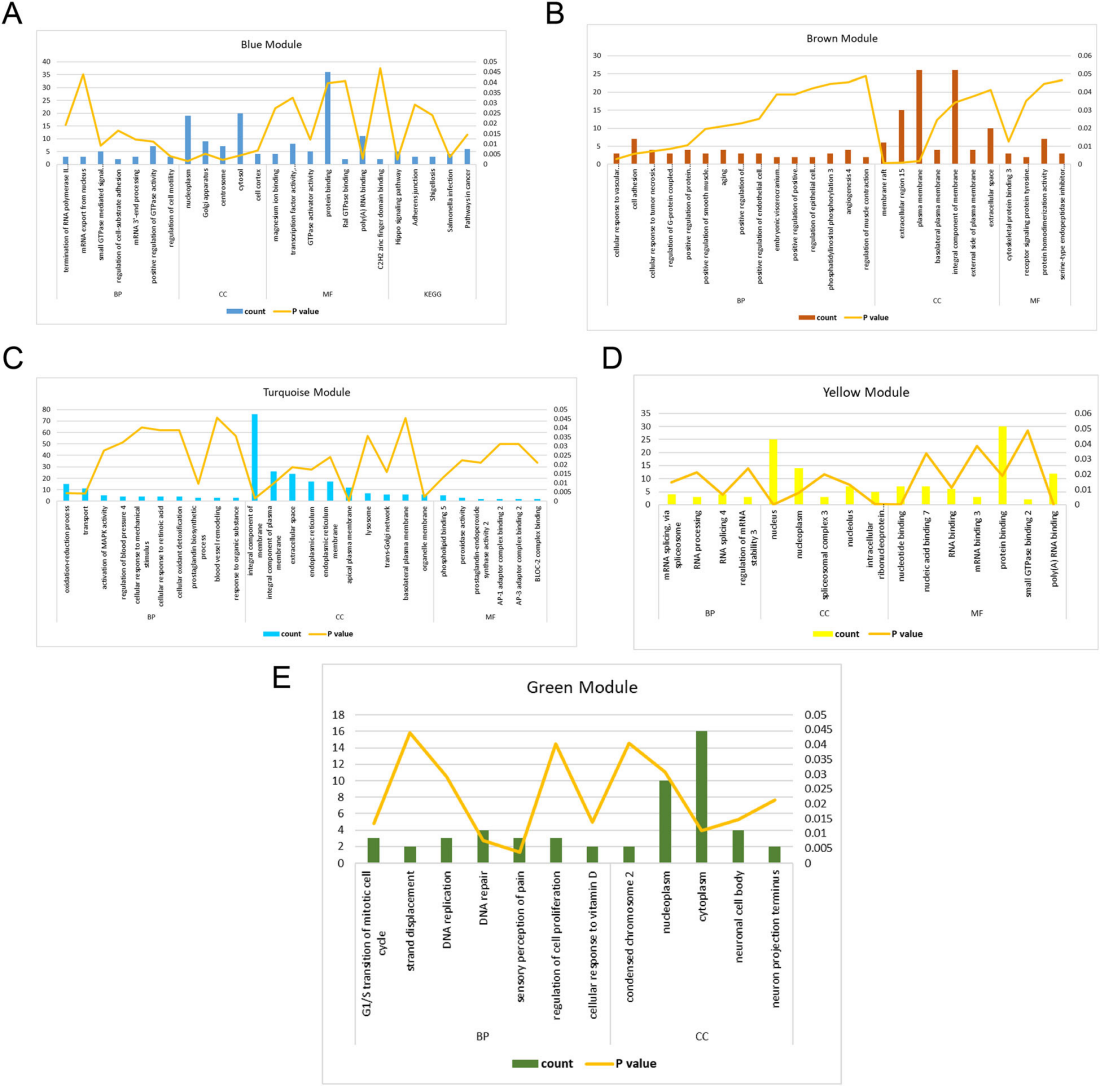
GO and KEGG enrichment analyses of the five modules. **A** The blue module. **B** The brown module. **C** The turquoise module. **D** The yellow module. **E** The green module. The X-axis represents enriched terms, the left Y-axis represents the number of enriched genes, and the right Y-axis represents the *P*-value. GO, Gene Ontology; KEGG, Kyoto Encyclopedia of Genes and Genomes; BP, biological process; CC, cellular component; MF, molecular function

To explore the functional roles of these different modules, the GO and KEGG analyses were further performed. The results showed that various biological processes may be involved in the pathogenesis of RIF, such as cell adhesion, regulation of cell motility, regulation of cell proliferation, activation of mitogen-activated protein kinase (MAPK) activity and cellular response to vascular endothelial growth factor (VEGF) stimulus (Fig. 4). The number of genes in the red module was too small to get functional enrichment results.

### Construction of ceRNA network

**Fig. 5:**
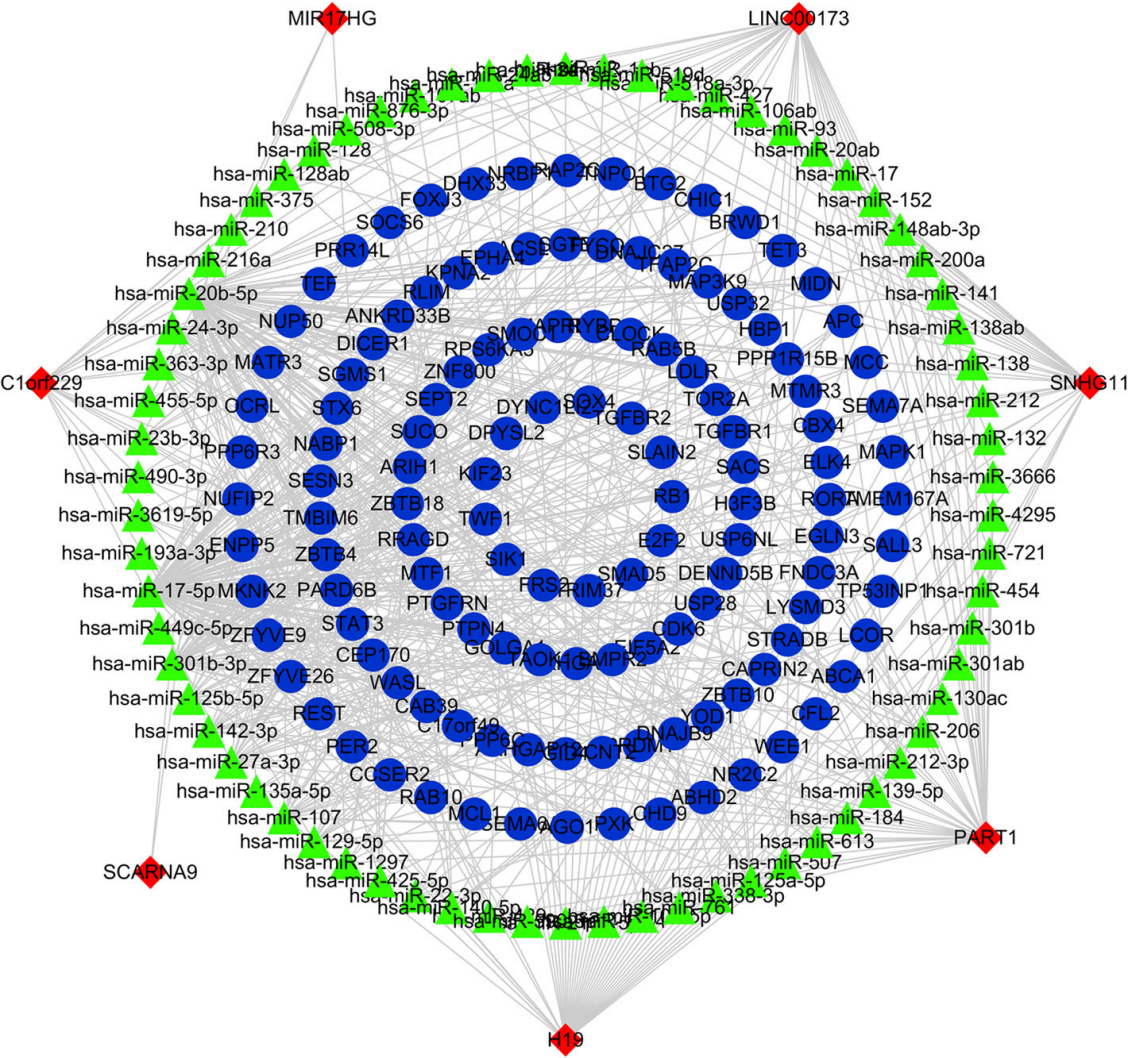
Construction of the competing endogenous RNA network. The red nodes represent lncRNAs, the green nodes represent miRNAs, and the blue nodes represent mRNAs. Only the nodes in the top 200 of degree ranking are presented

**Fig. 6:**
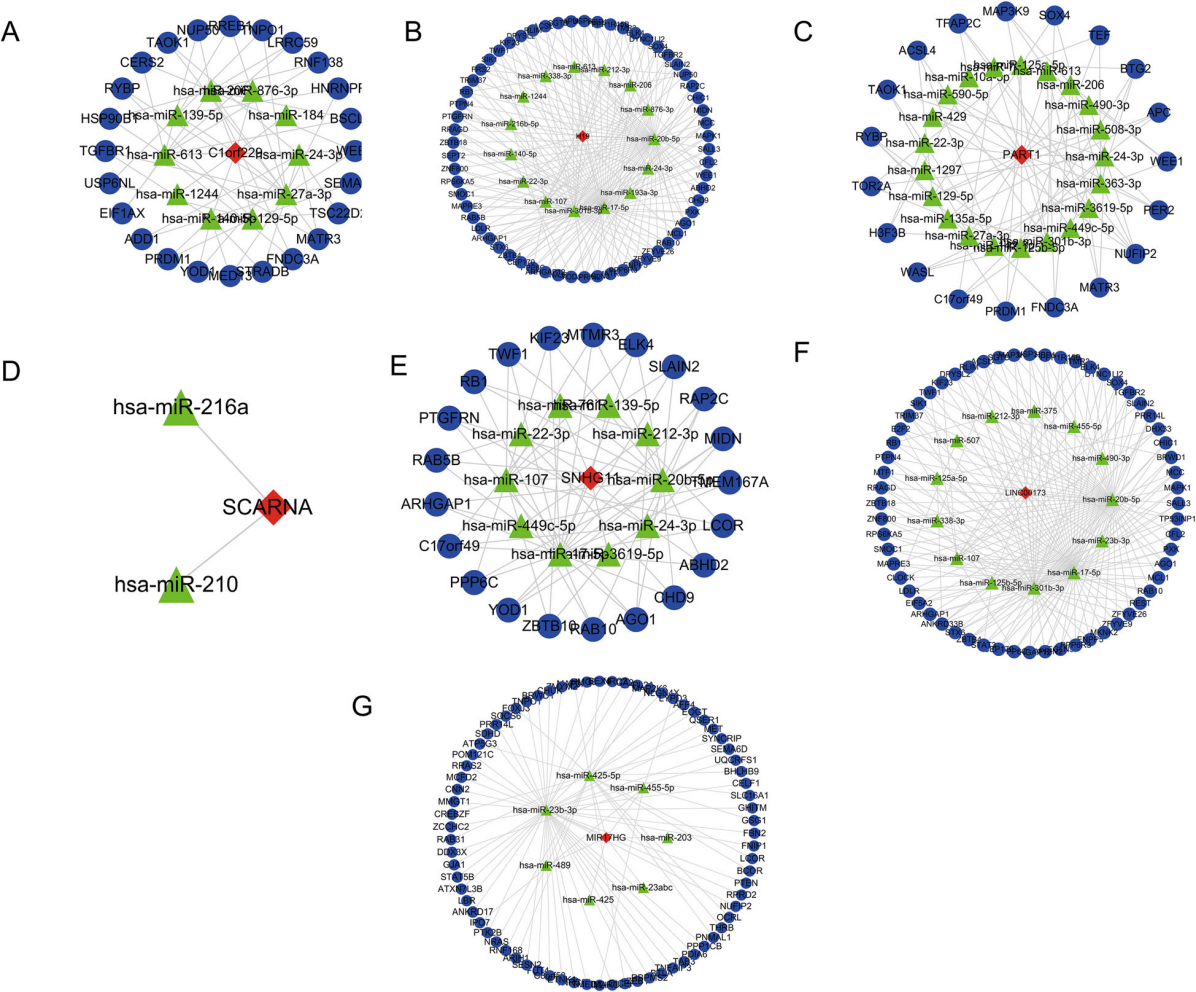
Competing endogenous RNA networks of seven hub lncRNAs. **A** C1orf229. **B** H19. **C** PART1. **D** SCARNA9. **E** SNHG11. **F** LINC00173. **G** MIR17HG. Nodes with different colors and shapes represent different RNA types

Based on the WGCNA co-expression modules, the interactions between lncRNAs/mRNAs and miRNAs were further predicted and a lncRNA-miRNA-mRNA ceRNA network was constructed (Fig. 5). Seven lncRNAs with the highest degree were selected as hub lncRNAs, including C1orf229, H19, PART1, SCARNA9, SNHG11, LINC00173 and MIR17HG (Fig. 6). The predicted lncRNA-miRNA and mRNA-miRNA pairs for WGCNA modules and hub lncRNA-related ceRNA networks are detailed in Tables S2 and  S3, respectively.

### Validation of hub lncRNAs

Figure S1 presents specifically the expression patterns of seven hub lncRNAs, which were further validated in another public dataset GSE71835. Consistently, the expression levels of PART1 and MIR17HG were significantly increased in RIF endometrium (Figure S2C and G), while those of H19 and LINC00173 were significantly decreased (Figure S2B and F). C1orf229, SCARNA9 and SNHG11 were also differentially expressed between RIF patients and controls, but the changes were not statistically significant (*P* > 0.05) (Figure S2A, D and E).

### Identification and validation of hub genes

**Fig. 7:**
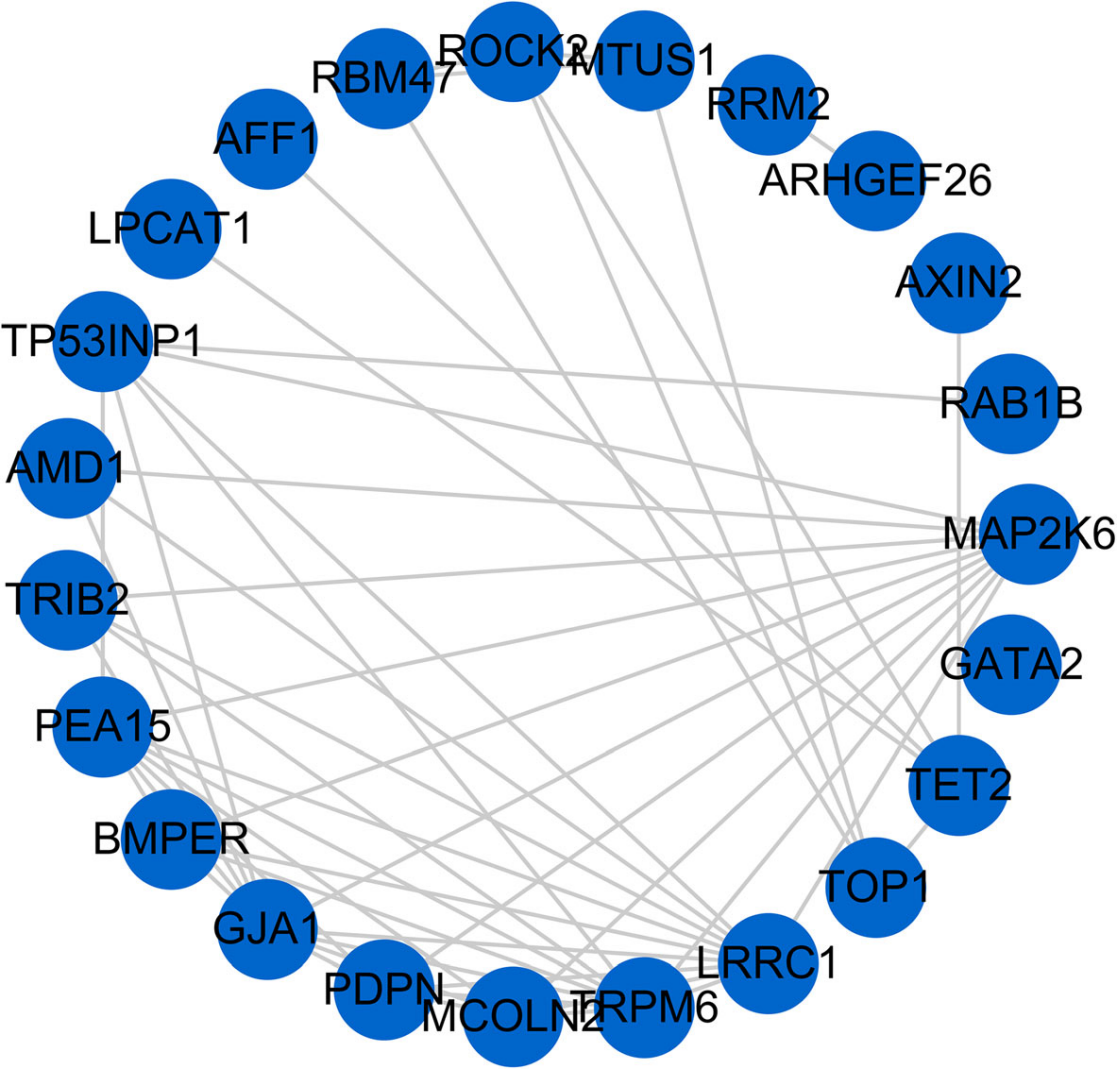
Co-expression network of overlapped genes in WGCNA and ceRNA analyses. The blue nodes represent mRNAs and the edges between nodes indicate co-expressions. WGCNA, weighted gene co-expression network analysis; ceRNA, competing endogenous RNA

To identify the RIF-related hub genes, we re-mapped the significantly DEMs in the ceRNA network to the WGCNA co-expression network. The final network consisted 23 nodes and 51 edges (Fig. 7). After sorting by the sum of Z-scores, a total of five genes were identified as hub genes, including TET2, GJA1, MAP2K6, LRRC1 and TRPM6. The details are presented in Table S4.

**Fig. 8:**
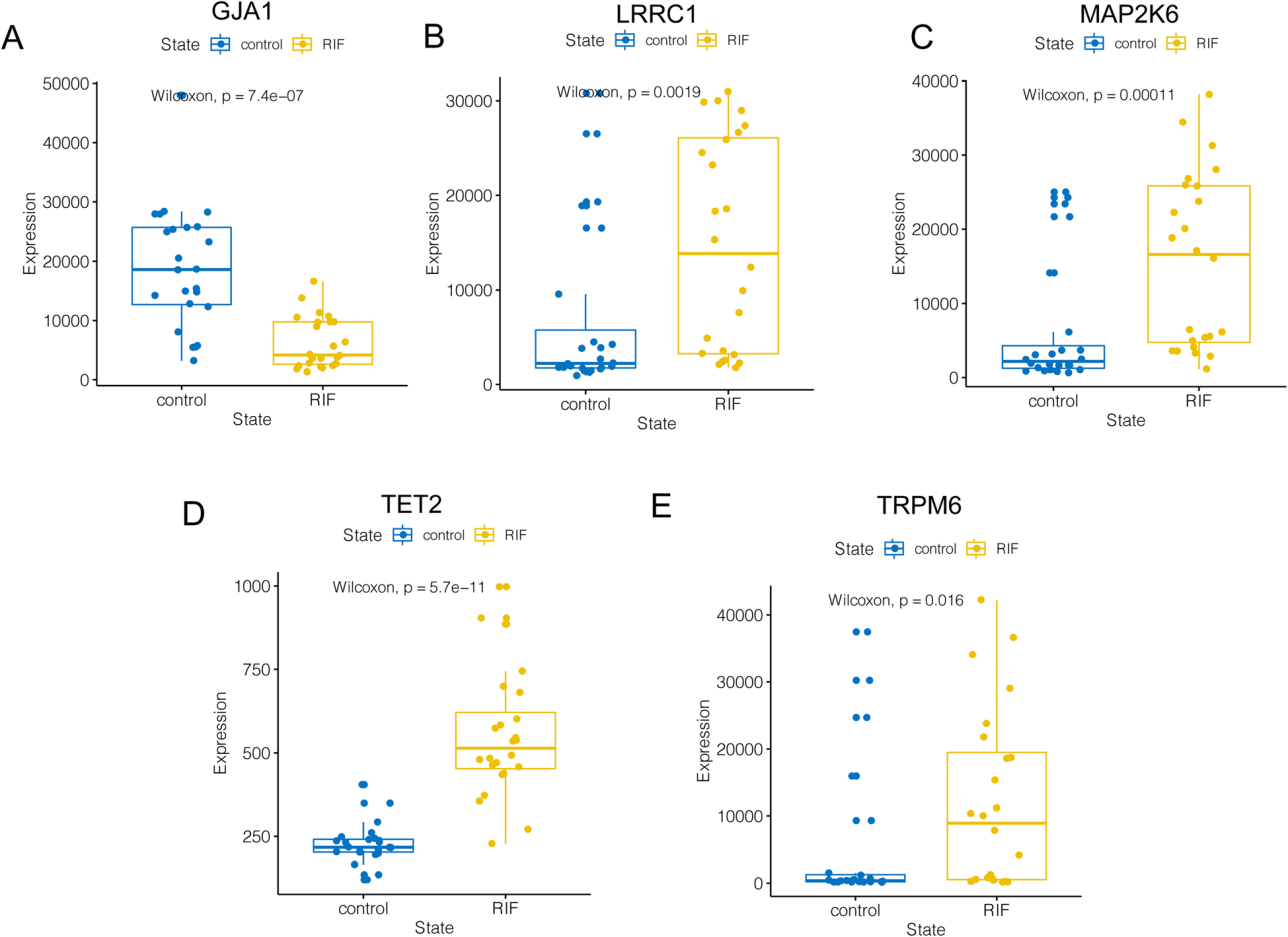
Differential expression of five hub genes in GSE111974 dataset. **A** GJA1. **B** LRRC1. **C** MAP2K6. **D** TET2. **E** TRPM6. RIF, recurrent implantation failure

**Fig. 9:**
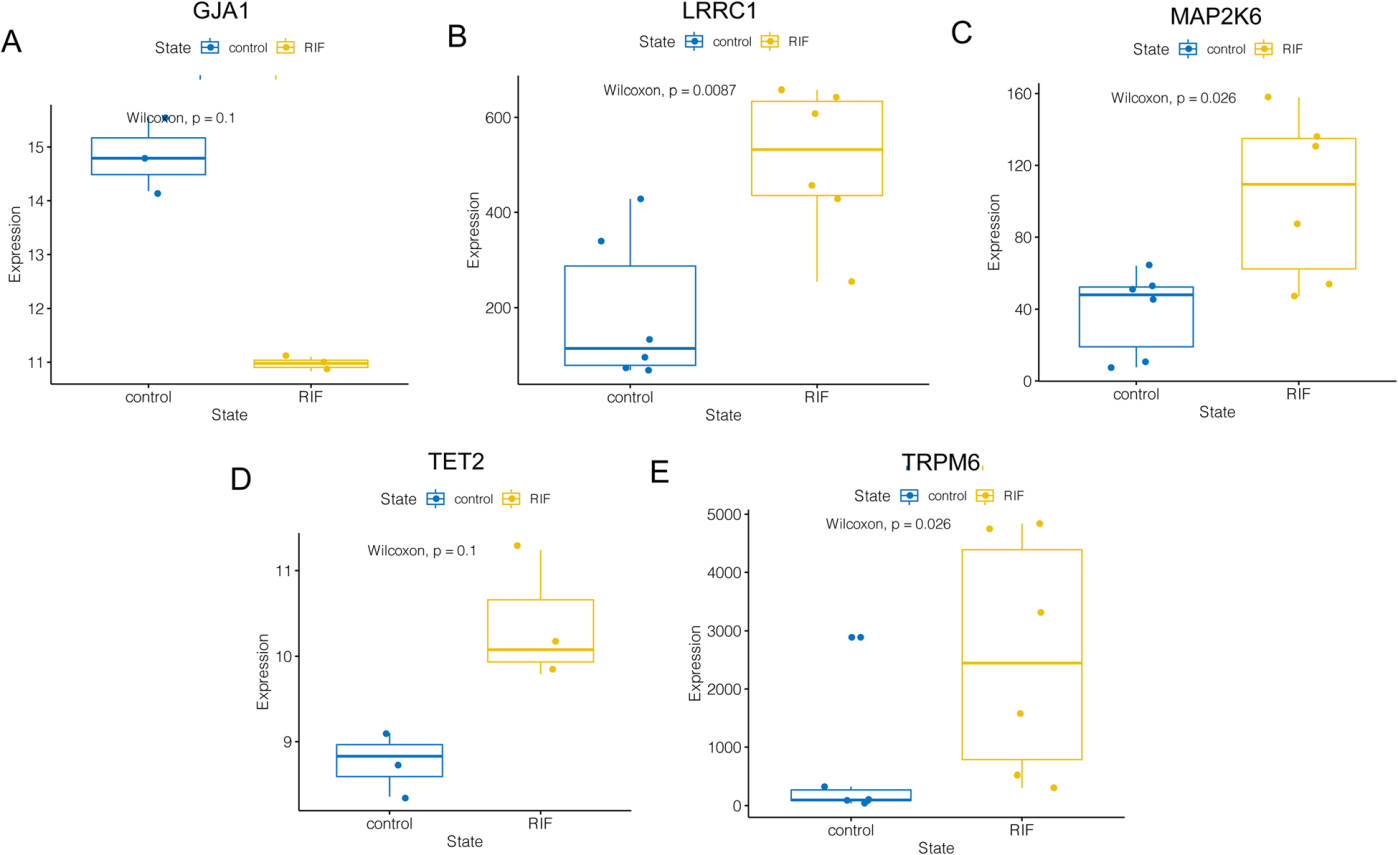
Differential expression of five hub genes in validation datasets GSE71835 and GSE103465. **A** GJA1. **B** LRRC1. **C **MAP2K6. **D** TET2. **E** TRPM6. RIF, recurrent implantation failure

According to the expression profile analysis of the GSE111974 dataset, TET2, MAP2K6, LRRC1 and TRPM6 showed significantly higher expression while GJA1 showed significantly lower expression in the RIF group compared with the control group (*P* < 0.05) (Fig. 8). Consistently, in the GSE71835 dataset, the expression of LRRC1, MAP2K6 and TRPM6 were also significantly higher in the endometrium of RIF patients (*P* < 0.05) (Fig. 9B, C and E), while data on GJA1 and TET2 could not be obtained. On the other hand, we found a trend for higher TET2 and lower GJA1 expression in the RIF group in the GSE103465 dataset, although no statistical differences were reached possibly due to the small sample size (both *P* = 0.1) (Fig. 9A and D). In general, the expression analyses of five hub genes in the external validation datasets were accordant with the results from the discovery dataset.

**Fig. 10:**
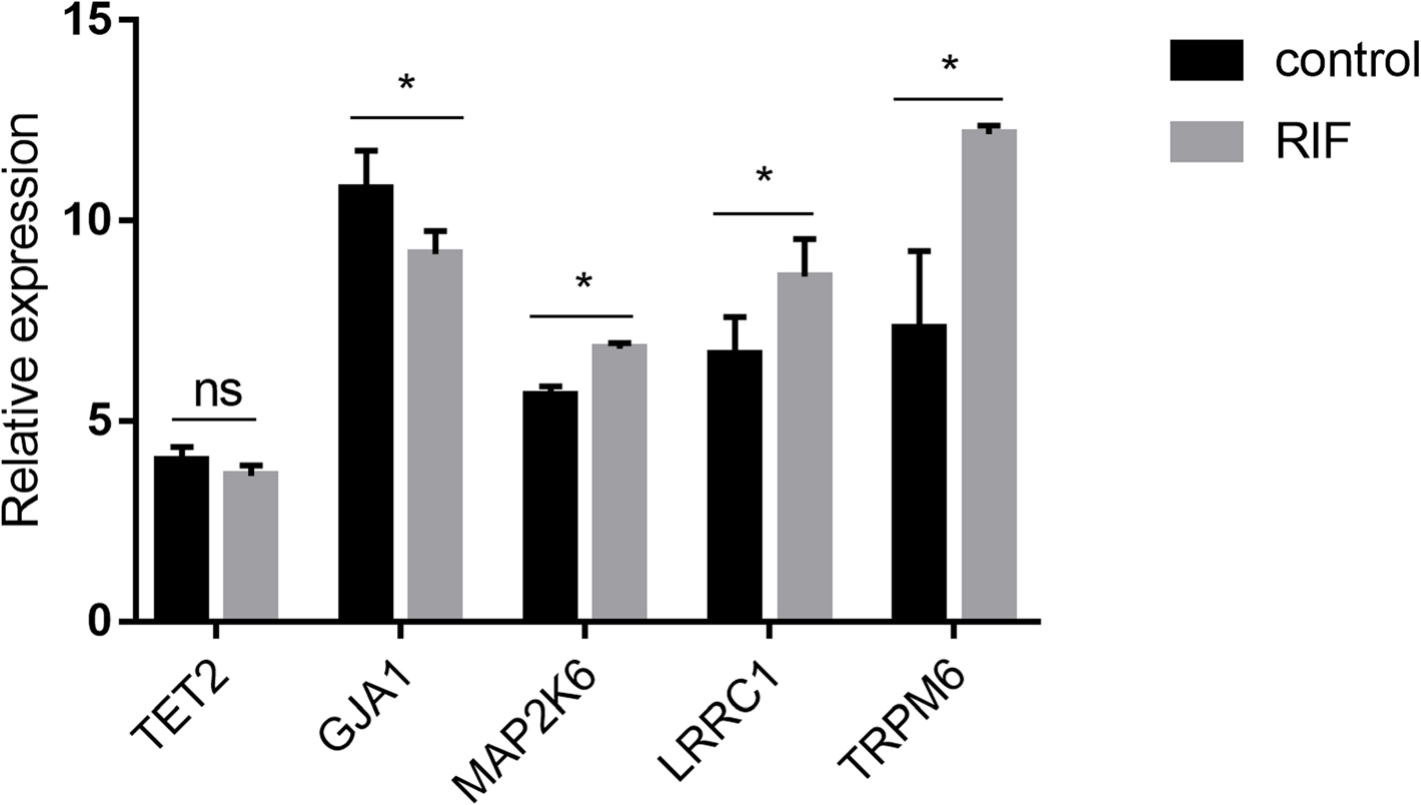
Validation of hub genes in the endometrium of RIF patients and controls by qRT-PCR. RIF, recurrent implantation failure. ns, not significant. * *P* < 0.05

The identified candidate genes were further assayed by qRT-PCR on a sample set of 10 RIF patients and 10 controls. Except for the number of embryo transfers (*P* < 0.001), other demographic characteristics were comparable between the two groups (all *P* > 0.05) (Table S5). As shown in Fig. 10, the mRNA expressions of GJA1, MAP2K6, LRRC1 and TRPM6 displayed the same trend as our *in silico* results, while no significant difference was detected in TET2.

## Discussion

As a complicated and poorly understood clinical disorder in IVF-ET cycles, RIF has brought significant burden to patients and therapeutic challenges to physicians. In this study, we applied an integrated bioinformatic approach to establish the RIF-related co-expression network and ceRNA network. In addition, five novel hub genes were identified and further validated as potential molecular targets underlying the development of RIF.

Six functional modules associated with RIF were built by WGCNA and analyzed in depth. In general, the GO and KEGG analyses showed that enriched genes and pathways were mainly involved in basic and essential biological events, such as cell adhesion, regulation of cell motility and regulation of cell proliferation, which were consistent with previous researches [17–21]. Indeed, in preparation for embryo implantation, the endometrial luminal epithelium must convert to an adhesive state for subsequent invasion of the hatched blastocyst [36]. Penetration of the trophoblasts also triggers a series of endometrial response called decidualization, which involves massive proliferation and differentiation of the stromal cells [37]. Moreover, activation of MAPK activity was found to be enriched in the turquoise module, which has been demonstrated to promote the proliferation of endometrial cells, the enhancement of uterine capacity and the maintenance of pregnancy [38, 39]. As one of the most important angiogenic factors, VEGF plays a vital role in decidual vascularization and placenta angiogenesis. Prior studies also suggest that VEGF polymorphisms are closely associated with the development of RIF [40, 41]. Accordingly, we found a significant enrichment of cellular response to VEGF stimulus in the brown module.

In the present study, seven hub lncRNAs with the highest degree were identified and verified in the ceRNA network, including C1orf229, H19, PART1, SCARNA9, SNHG11, LINC00173 and MIR17HG. Among them, the lncRNA H19, which is mainly located in the cytoplasm with a length of approximately 2.3 kb, plays diverse roles in multiple physiological and pathological processes [11, 12]. In human endometrium, H19 is confined to the endometrial stroma and expressed in a menstrual cycle-dependent manner, with peaks reached during the late proliferative stage [42]. Previous studies have shown that the expression of H19 was significantly decreased in women with unexplained infertility [43], spontaneous abortion [44], endometriosis [45] as well as RIF [46]. Acting as a molecular sponge, H19 downregulation could increase the activity of miRNA let-7 and thus inhibit its downstream target ITGB3 at the post‐transcriptional level, which further contributes to impaired adhesion and invasiveness of extravillous trophoblast [47]. In addition, aberration of the H19/let‐7/IGF1R regulatory pathway has been associated with a negative impact on stromal cell proliferation and may thus decrease endometrial receptivity for pregnancy [45]. Overexpression of PART1, SNHG11, LINC00173 and MIR17HG is widely involved in tumorigenesis and malignant progression of several types of cancer, such as glioma, breast cancer, lung cancer and colorectal cancer [48–52]. Potential mechanisms have been focused on the promotion of cell proliferation, migration, invasion and epithelial-mesenchymal transition, which are also important for the establishment of receptive endometria and the process of embryo implantation [36, 37]. Therefore, these lncRNAs may regulate the development of RIF as well, although no relevant findings have been reported for confirmation thus far. With regard to molecular mechanisms of C1orf229 and SCARNA9, the scarce studies at present require the need for further investigations.

Co-analysis of the WGCNA network and the lncRNA-related ceRNA network identified five novel hub genes in RIF, namely TET2, GJA1, MAP2K6, LRRC1 and TRPM6. The differential expression was also in general consistency among the discovery dataset GSE111974, the validation datasets GSE71835/GSE103465 and qRT-PCR analysis using *in vivo* samples. Dysregulation of TET2 is commonly observed in myeloid and lymphoid malignancies [53]. As one of the three proteins in the TET (ten-eleven translocation) family, TET2 is an α-ketoglutarate-dependent dioxygenase that enables the conversion of 5-methyl-cytosine to 5-hydroxymethyl-cytosine and promotes DNA demethylation [54]. In this regard, the increased TET2 in RIF might be associated with hypomethylation of CYP19A1, ESR2 and SF-1 genes, thus leading to an estradiol-rich endometrial environment and defective decidualization [55–57]. Connexin 43 (Cx43), the product of GJA1 gene, is a constitutive part of gap junction intercellular communication and predominantly expressed in endometrial stroma [58]. Ablation of Cx43 expression has been reported to suppress proliferation, impair differentiation and induce apoptosis of endometrial stromal cells [58, 59]. The production of key angiogenic factors (e.g., VEGF) was also significantly reduced, whereas the secretion of inflammatory mediators was increased [60]. Collectively, there disruptions could result in an unreceptive endometrium with embryo growth arrest and early pregnancy loss [58]. MAP2K6 is an upstream kinase of the p38 MAPK signal cascade activated under the status of inflammation and stress. Previous studies have demonstrated that MAP2K6 plays crucial roles in various biological processes, such as cell cycle regulation, transcription and apoptosis [61]. In Ishikawa endometrial cell lines, transfection of MAP2K6 could stimulate the estrogen-mediated transcription and proliferation through selective activation of p38 MAPK, which further phosphorylates and potentiates the p160 coactivator glucocorticoid receptor-interacting protein 1 [62]. Therefore, MAP2K6 upregulation in RIF may augment estrogen action in the secretory endometrium and thus impair the progesterone-dependent decidualization process. LRRC1 belongs to the LAP protein family and is vital for the establishment and maintenance of apical-basal cell polarity. Throughout the proliferative phase of the menstrual cycle, human endometrial luminal epithelium also displays a distinct polarized organization. However, during the secretory phase, loss of polarity must occur to overcome mutual repulsion and thus facilitate the interaction with embryos for implantation [63, 64]. In this regard, high LRRC1 expression may impede cellular rearrangements for polarity modulation and consequently lead to the development of RIF. As the member of TRP (transient receptor potential) superfamily, TRPM6 acts as an important ion channel for Ca^2+^/Mg^2+^ influx that transduces environmental stimuli into cellular responses [65]. In human endometrium, the expression of TRPM6 increases during the follicular phase, peaks in the early luteal phase, but decreases sharply in the late luteal phase when progesterone levels are high [66, 67]. This specific downregulation around the window of implantation suggests that TRPM6 might be important to necessitate functional progesterone receptors and confer endometrial receptivity. Contrarily, significant upregulation of TRPM6 was found in women with RIF, further implicating its possible role in mediating decidualization and embryo implantation. Overall, these hub genes provide novel insights into the pathogenesis of RIF and deserve more exploration on the detailed mechanisms.

Some limitations should be acknowledged of the current study. On the one hand, the sample size of our analyzed dataset was relatively small, with only 24 RIF patients and 24 control women enrolled. Therefore, the results may not be robust enough and more studies with larger sample size should be carried out in the future. On the other hand, although the expression of hub genes was validated in endometrial tissue samples from IVF-ET patients, we did not assess further their biological functions predicted by bioinformatic tools. In this regard, we have obtained the hospital’s ethical approval for study and several *in vitro* experiments are now undergoing for more investigation.

## Conclusions

In summary, our study provided a comprehensive bioinformatic analysis of the WGCNA co-expression network and lncRNA-associated ceRNA network for RIF. We identified five novel hub genes that showed significantly differential expression in RIF and may play crucial roles in endometrial receptivity. Our findings could be helpful to further understand the molecular mechanisms for RIF pathogenesis, and also lay a foundation for the discovery of potential diagnostic and therapeutic targets.

## Supplementary Information

**Additional file 1: Figure S1. **Differential expression of seven hub lncRNAs in GSE111974 dataset. (A) C1orf229. (B) H19. (C) PART1. (D) SCARNA9. (E) SNHG11. (F) LINC00173. (G) MIR17HG. RIF, recurrent implantation failure.

**Additional file 2: Figure S2. **Differential expression of seven hub lncRNAs in validation dataset GSE71835. (A) C1orf229. (B) H19. (C) PART1. (D) SCARNA9. (E) SNHG11. (F) LINC00173. (G) MIR17HG. RIF, recurrent implantation failure.

**Additional file 3: Table S1.** List of primers used for qRT-PCR.

**Additional file 4: Table S2.** Predicted lncRNA-miRNA and mRNA-miRNA pairs for WGCNA modules. WGCNA, weighted gene co‐expression network analysis.

**Additional file 5: Table S3.** Predicted lncRNA-miRNA and mRNA-miRNA pairs for the seven hub lncRNA-related ceRNA networks. ceRNA, competing endogenous RNA.

**Additional file 6: Table S4. **Topological characteristics of 23 genes overlapped in WGCNA and ceRNA networks. WGCNA, weighted gene co‐expression network analysis; ceRNA, competing endogenous RNA.

**Additional file 7: Table S5.** Demographic characteristics of recruited controls and RIF patients. RIF, recurrent implantation failure.

## Data Availability

The datasets supporting the conclusions of this article are included within the article and its additional files.

## Abbreviations

BC: Betweenness centrality
CC: Closeness centrality
ceRNA: Competing endogenous RNA
Cx43: :Connexin 43
DAVID: Database for Annotation, Visualization and Integrated Discovery
DC: Degree centrality
DELs: Differentially expressed long non-coding RNAs
DEMs: Differentially expressed messenger RNAs
GEO: Gene Expression Omnibus
GO: Gene Ontology
IVF-ET: *In vitro* fertilization-embryo transfer
KEGG: Kyoto Encyclopedia of Genes and Genomes
LncRNA: Long non-coding RNA
mRNA: Messenger RNA
MAPK: Mitogen-activated protein kinase
miRNA: Micro RNA
qRT-PCR: Quantitative real-time polymerase chain reaction
RIF: Recurrent implantation failure
TET: Ten-eleven translocation
TOM: Topological overlap matrix
VEGF: Vascular endothelial growth factor
WGCNA: Weighted gene co-expression network analysis
