# Supplementary figures and images for: Construction of lncRNA-related competing endogenous RNA network and identification of hub genes in recurrent implantation failure
Source: Reprod Biol Endocrinol. 2021 Jul 9;19:108. doi: 10.1186/s12958-021-00778-1 (PMC8268333; doi:10.1186/s12958-021-00778-1)

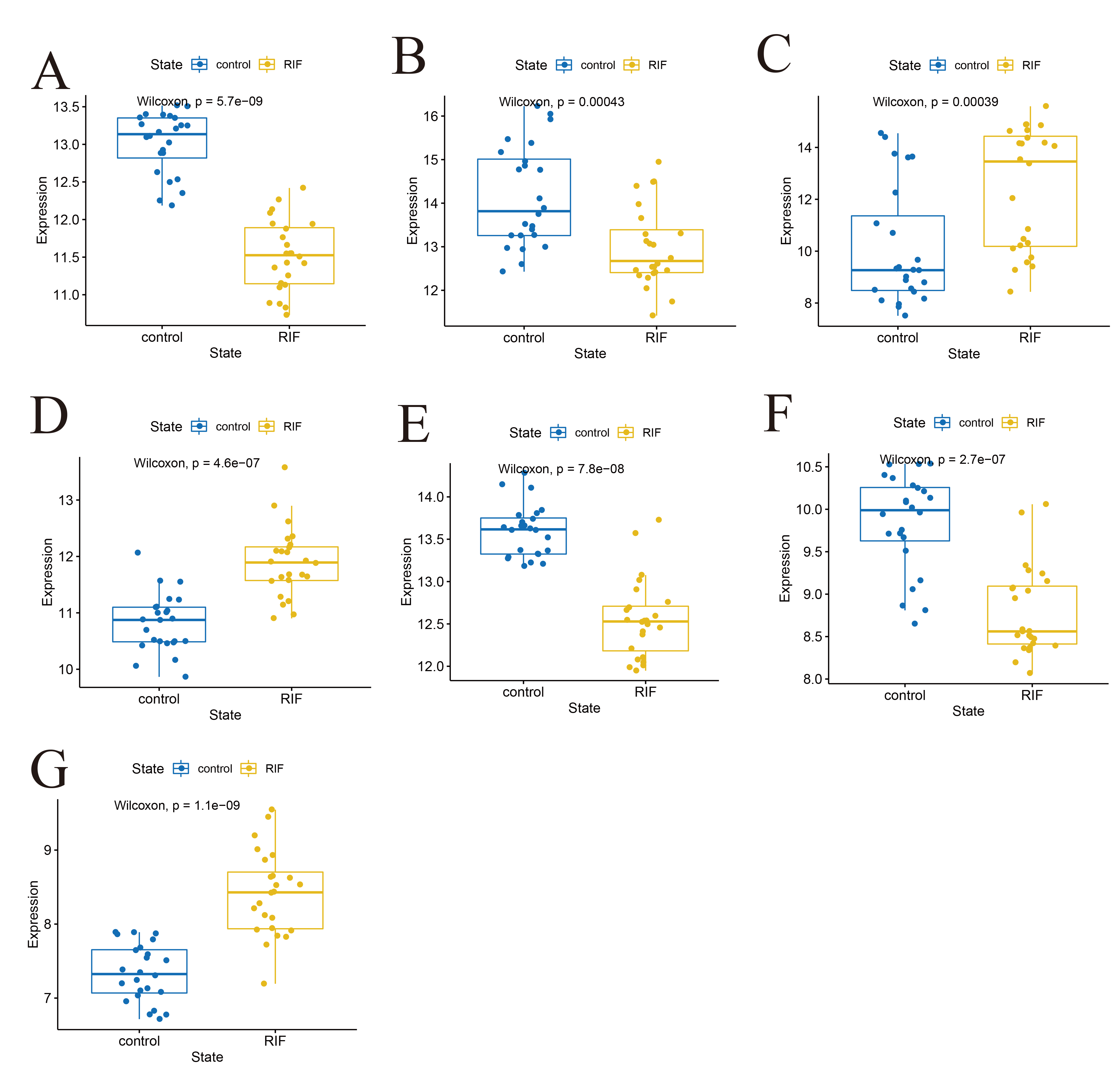

Supplement: Supplementary file 1 — Additional file 1: Figure S1. Differential expression of seven hub lncRNAs in GSE111974 dataset. (A) C1orf229. (B) H19. (C) PART1. (D) SCARNA9. (E) SNHG11. (F) LINC00173. (G) MIR17HG. RIF, recurrent implantation failure. [file 12958_2021_778_MOESM1_ESM.tif]

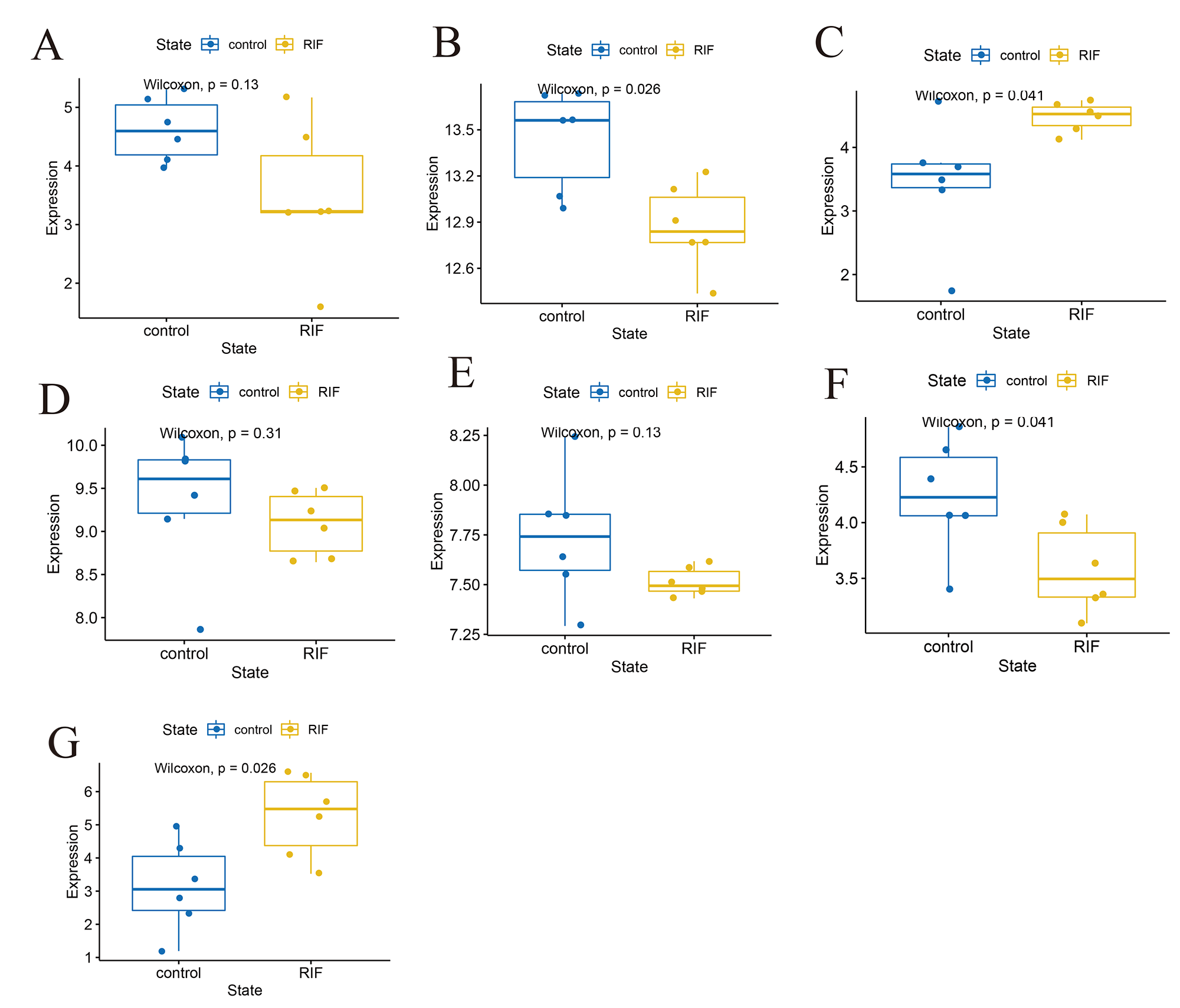

Supplement: Supplementary file 2 — Additional file 2: Figure S2. Differential expression of seven hub lncRNAs in validation dataset GSE71835. (A) C1orf229. (B) H19. (C) PART1. (D) SCARNA9. (E) SNHG11. (F) LINC00173. (G) MIR17HG. RIF, recurrent implantation failure. [file 12958_2021_778_MOESM2_ESM.tif]
