# Supplementary material for: Construction of lncRNA-related competing endogenous RNA network and identification of hub genes in recurrent implantation failure
Source: Reprod Biol Endocrinol. 2021 Jul 9;19:108. doi: 10.1186/s12958-021-00778-1 (PMC8268333; doi:10.1186/s12958-021-00778-1)
Supplement: Supplementary file 3 — Additional file 3: Table S1. List of primers used for qRT-PCR. [file 12958_2021_778_MOESM3_ESM.docx]

Table S1. List of primers used for qRT-PCR.

| Genes | Primer forward (5’-3’) | Primer reverse (5’-3’) |
| --- | --- | --- |
| TET2 | CCCACAGAGACTTGCACAACAT | CTGGCTCTGCTAACATCCTGAC |
| GJA1 | TCTCTCATGTGCGCTTCTGG | TGACACCATCAGTTTGGGCA |
| MAP2K6 | AGCCACGGACTTGACTGGTTGACC | GGACTCCTCGTATGCCTCGCCATT |
| LRRC1 | TCCTTACCAAAAGAGATCGG | GGTAGATGCAGCAACCTGT |
| TRPM6 | CCACCAATACCCTGGAAGAA | AGGAGTTGCAGCGATGTTTT |
| β-actin | CGCGAGAAGATGACCCAGAT | ACAGCCTGGATAGCAACGTA |
