# Supplementary material for: Construction of lncRNA-related competing endogenous RNA network and identification of hub genes in recurrent implantation failure
Source: Reprod Biol Endocrinol. 2021 Jul 9;19:108. doi: 10.1186/s12958-021-00778-1 (PMC8268333; doi:10.1186/s12958-021-00778-1)
Supplement: Supplementary file 7 — Additional file 7: Table S5. Demographic characteristics of recruited controls and RIF patients. RIF, recurrent implantation failure. [file 12958_2021_778_MOESM7_ESM.docx]

Table S5. Demographic characteristics of recruited controls and RIF patients.

|  | Control (*n* = 10) | RIF (*n* = 10) | *P*-value |
| --- | --- | --- | --- |
| Age (years) | 30.1 ± 2.0 | 29.5 ± 3.4 | 0.483 ^a^ |
| Body mass index (kg/m^2^) | 21.4 ± 1.9 | 21.3 ± 1.9 | 0.814 ^a^ |
| Infertility duration (years) | 3.2 ± 1.5 | 3.5 ± 1.3 | 0.529 ^b^ |
| Basal follicle-stimulating hormone (IU/L) | 7.2 ± 0.6 | 7.4 ± 0.9 | 0.579 ^b^ |
| Basal luteinizing hormone (IU/L) | 5.0 ± 0.5 | 4.7 ± 0.6 | 0.428 ^a^ |
| Basal estradiol (pg/mL) | 36.6 ± 10.5 | 36.9 ± 9.9 | 0.900 ^a^ |
| Anti-Müllerian hormone (ng/mL) | 5.4 ± 0.5 | 5.1 ± 0.6 | 0.689 ^a^ |
| Antral follicle count | 12.9 ± 3.4 | 11.9 ± 3.0 | 0.437 ^a^ |
| Endometrial thickness (mm) | 11.3 ± 1.8 | 10.7 ± 1.8 | 0.315 ^b^ |
| Number of embryo transfers, *n* (%) |  |  | <0.001 ^c^ |
| 1 | 8 (80.0) | - |  |
| 2 | 2 (20.0) | - |  |
| 3 | - | 4 (40.0) |  |
| 4 | - | 4 (40.0) |  |
| 5 | - | 1 (10.0) |  |
| 6 | - | 1 (10.0) |  |

Data are presented as mean ± standard deviation or number (proportion).

^a^ Student’s *t* test.

^b^ Mann-Whitney *U* test.

^c^ Fisher’s exact test.
